# Versatile Movements of Liquid Metal Droplet under Electrostatic Actuation in Alkaline Solutions

**DOI:** 10.3390/ma13092122

**Published:** 2020-05-03

**Authors:** Qingming Hu, Tianyi Jiang, Hongyuan Jiang

**Affiliations:** 1 School of Mechatronics Engineering, Harbin Institute of Technology, Harbin 150001, China; jty_hit@hit.edu.cn; 2 School of Mechatronics Engineering, Qiqihar University, Qiqihar 161006, China

**Keywords:** droplet manipulation, AC electrical filed, surface tension, continuous electrowetting, galinstan liquid metal

## Abstract

The gallium-based eutectic liquid metal alloys exhibit unique properties of deformability, excellent electrical conductivity and low vapour pressure. The liquid metal-based circuits’ element or actuator have drawn considerable attention in stretchable electronics and microelectromechanical (MEMS) actuators. Yet, the motion of the liquid metal within the electrolyte needs to be precisely regulated to satisfy application requirements. Herein, we investigated the locomotion of liquid metal within the alkaline aqueous solution under electrostatic actuation. The relationship between the travelling speed of the liquid metal slug and the relative influential parameters, such as the voltage amplitude and frequencies of the applied electric field, electrolyte concentration, electrodes distance and the liquid metal volume, were experimentally characterized. A travelling speed up to 20.33 mm/s was obtained at the applied voltage of 4 Vpp at 150 Hz at 6 V DC offset. Finally, the frequency-dependent liquid metal marble movements were demonstrated, namely oscillation and forward locomotion while oscillating. The oscillation frequency was determined by the frequency of the applied alternate current (AC) signal. The remarkable transportation and oscillating characteristic of the liquid metal marble under the electrostatic actuation may present potentials towards the development of flexible electronics and reconfigurable structures.

## 1. Introduction

Due to various remarkable advantages, such as reduced agent consumption, smaller analysis volume, less reaction time and increasing device portability, the droplet-based microfluidic has recently played increasing important in the fields of microelectromechanical systems (MEMS) and lab-on-a-chip microfluidic devices [1,2,3]. Some other micro chemical analysis systems also have urgent demand for micro fluid handling, such as fluid mixing and fluid pumping [4]. Particularly, controllable manipulation of discrete droplets in the microchannel has been extensively investigated by the research community as it could enable some complex microfluidic processing to be accomplished in the integrated microfluidic system without using conventional external components, mainly including droplet transporting, coalescing, splitting and mixing [5]. The droplet of unique physicochemical properties could be adopted as a relevant electrical, optical or thermal component. Meanwhile, the previous studies also indicated that the complicated and interesting fluid flow behavior was induced around the droplet during the processing of droplet manipulation, which could be used for liquid handling in the microfluidic devices, for example, preprocessing or sample dilution, and mixing of analytes and reagents in the continuous flow system [6]. The geometric curvature of the membrane under the electrostatic field was introduced to membrane MEMS devices, and the numerical model concerning the curvature of the membrane and the electrical magnitude was investigated [7,8,9]. Therefore, due to the unique advantages, the droplet-based microfluidic technique and its potential application in MEMS, due to the avoidance of mechanical contact, it is of vital importance to reveal the flow behavior and to manipulate individual aqueous droplets immersed in an immiscible fluid in the microfluidic channel.

To date, various approaches have been developed for manipulating droplets. With the shrinking down of the droplet size, the surface tension becomes the dominant force in microscale compared with other forces, due to the large surface area to volume ratio, such as gravitational force and inertial force. The ability to control the surface tension provides an alternative powerful approach to actuate the droplet, which has been adopted as the driving force in micro total analysis systems. Several approaches have been proposed to manipulate microdroplets within electrolytes using the variation of interfacial tension above the droplet surface according to the droplet actuation mechanisms, mainly including surfactants [10,11,12], thermocapillary [13,14,15], magnetic force [16,17,18], and electrostatic actuation [19,20,21]. The surface tension difference induced by local surface temperature gradients using the thermocapillary effect-driven mechanism could adjust the surface tension along the droplet, leading to the liquid moving toward the colder regions. However, considerable power are required to generate enough surface tension difference to transfer the droplet, leading to rapid liquid evaporation and not suitable for some highly volatile liquids. The superparamagnetic beads were explored to modify the droplet properties and the magnetic force was allowed for accomplishing droplet manipulation, which could be used for cell lysis and polymerase chain reaction (PCR) amplification. Among them, the electrostatic method was introduced to manipulate the droplet based on the electrical control of the surface tension, which involves no moving mechanical parts and has attracted great attention from the research community. Dielectrophoresis is one of the commonly used actuation manipulation methods for moving droplets. With a non-uniform electric field implemented onto the droplet, a DEP force can be produced and we can transfer the droplet to the designated location without contacting the electrodes, which is beneficial for less contamination between the droplets and the electrodes [22]. However, as the DEP force largely depends on the dielectric property of the droplet, it often requires higher voltage to generate bigger droplet velocity. 

Additionally, with so many extraordinary physicochemical characteristics, such as high surface tension, high conductivity and thermal conductivity, room temperature liquid metal has drawn considerable attention in various electrochemical and industrial applications [23,24,25,26]. While in comparison with conventional laboratory experimentally used mercury, the gallium-based eutectic alloys liquid metal characterized by low vapor pressure, high mobility and deformability, and low toxicity enabled it as functional material in various applications of MEMS actuators [27,28,29,30], heat exchanger [31,32] and reconfigurable electronic devices [33,34,35]. Under the most aforementioned occasions, it required to precisely modulate the motion of liquid metal in continuous flow system, for example, the microvortex was induced around the liquid metal droplet to generate chaotic advection to mix the neighboring incoming fluids with the liquid metal actuator activated by an external exciting signal [28]. Liquid metal actuation by controlling the surface tension is effective in the liquid handling microsystem. The magnetic force and the electric potential could be utilized to alter the surface tension and the resultant fluid flow was generated. Using the magnetic force driving mechanism, the liquid metal droplet was modified by spraying or sprinkling ferromagnetic nanoparticles on the droplet surface, and then, the droplet motion and deformation were adjusted by the external magnetic field. Zhang [36] presented a method to modify the liquid metal with copper-iron magnetic nanoparticles and experimentally investigated the movement of functional liquid metal droplets with respect to the intensity of the applied magnetic field, indicating that the liquid metal droplet after surface modification had good deformability and could be flexibly actuated within a certain space with magnetic field. Although the desirable shape and controllable self-locomotion of liquid metal could be flexibly realized by utilizing magnetic field, the electroplating process for the droplet surface modification seemed to be intricate. Meanwhile, the density and size distribution of the additive nanoparticles would sometimes influence the intrinsic properties and the driving performance of the liquid metal by decreasing the surface fluidity and deformability. Recently, the electric field was introduce to regulate the surface tension by creating a local surface-tension variation in the two-phase fluids interface between the two neighboring immiscible conductive fluid flow, which would control the motion and deformation of the liquid metal droplet and has been known as electrocapillary actuation. Unlike the traditional pumping effect requiring moving parts, electrowetting is a popular electrocapillary phenomenon for transferring droplets, in which the interface tension between the driving electrode and conducting liquid phase was electrostatic-adjusted through the external potential gradient acting on the liquid droplet. With a thin dielectric layer between the conductive liquid and the embedded planar electrode, electrowetting on dielectric (EWOD) could prevent electrolysis with AC signals applied. Therefore, the electrowetting actuation of liquid metal has gained relative importance in reconfigurable components due to its rapid switching response [37,38,39,40,41]. Wan [41] presented an electrowetting-actuated micromirror device with a liquid metal droplet embedded between the substrate and parylene/Teflon Coated indium tin oxide (ITO) glass substrate, in which the reflective area was adjusted by the external exciting signal. Although the EWOD actuation mechanism has the merit of no moving parts, no heating of the transporting medium and lower power consumption, the fabrication of a dielectric-coated electrode such as ITO thin film in the microchannel is complicated. Meanwhile, it always required several electrodes and intricate control circuit to accomplish long distance transportation. While recently, another surface tension-induced mechanism, the continuous electrowetting, was introduced to actuate the discrete liquid metal by tailoring the liquid–liquid interfacial tension [42,43,44,45]. Gough [45] present a reconfigurable radio frequency device with a non-toxic liquid metal slug within the fluidic microchannel as a tuning element by continuous electrowetting actuation. 

As the liquid handling was of vital importance in the MEMS device, we herein demonstrated an open channel system with the non-toxic galinstan liquid metal slug electrostatically actuated along the channel. The transportation of the liquid metal was controlled by the external applied electrical field. We investigated the effect of the voltage amplitude and exciting frequency, electrolyte concentration and droplet volume on the pumping velocity of the liquid metal. We also determined the dependence of droplet pumping mode on the frequency of the input AC signal, including unidirectional pumping, oscillation and forward locomotion while oscillating.

## 2. Materials and Theory

### 2.1. Experimental Design and Chip Fabrication

**Figure 1:**
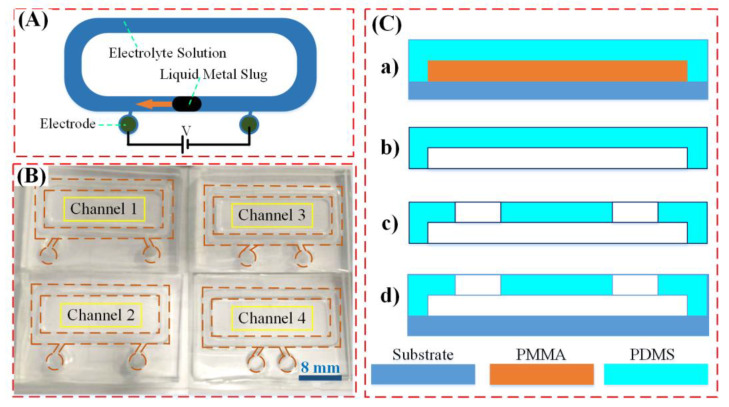
Chip design and fabrication processes of the liquid metal-based actuation device. (**A**) The chip structure of the liquid metal actuation device. (**B**) Image of the PDMS channel used for liquid metal pumping. (**C**) The fabrication process of the presented device.

The chip structure and the fabrication processes of the liquid metal-based microfluidic device were depicted in Figure 1, which incorporated a liquid metal slug confined in the alkaline solution-filled channel and two graphite electrodes located on both ends of the liquid metal. A close-loop polydimethylsiloxane (PDMS) channel was located on the glass slide, with the width and height of 2 and 2.65 mm, respectively. The galinstan liquid metal (purchased from Sigma) was utilized as the core of the chip for pumping the alkaline solution, and the droplet diameter of 2.5 mm was adopted in the experiments. The two graphite electrodes separated by a certain distance ranging from 4 to 16 mm with a increment of 4 mm on both sides of the chip (as shown in Figure 1B), which was excited by the signal generator (TGA 12104, TTI, Cambs, UK). The top view images and pumping videos were recorded by a digital camera (Retiga-2000R, QImaging, Surrey, BC, Guildford, Canada), and then, the video was processed by Image J software (version 1.44p, NIH, Bethesda, MD, USA).

The fabrication processes of the channel were illustrated in Figure 1C, with the PDMS elastomer prepared by conventional soft lithographic technique. The thermoplastic polymer polymethyl methacrylate (PMMA) was firstly tailored by a laser cutter (K3020, Huitian, Dongguan, China) according to the two-dimensional layout design. Then, the PMMA was adhered onto a glass substrate with glue to serve as the channel mold. The PDMS (Sylgard 184, Dow Corning Inc., Midland, MI, USA) solution, mixed at a weight ratio of 10:1 with a curing agent (Sylgard 184 A&B, Dow Corning, Inc.), was poured over the master structure after being vacuumized for 30 min in a vacuum chamber to remove the air bubbles to create an elastomeric mold. The solid-state PDMS mixture was released from the mold after it was pre-cured in the oven at 80 °C for 3 h. With the PDMS layer being plasma treated by oxygen plasma (ZEPTO, Diener, Ebhausen, Germany), it was adhered to another glass substrate to form the chip. Finally, the chip was put into the oven to post-cure it for 2 h at 65 °C.

### 2.2. Continuous Electrowetting Actuation

**Figure 2:**
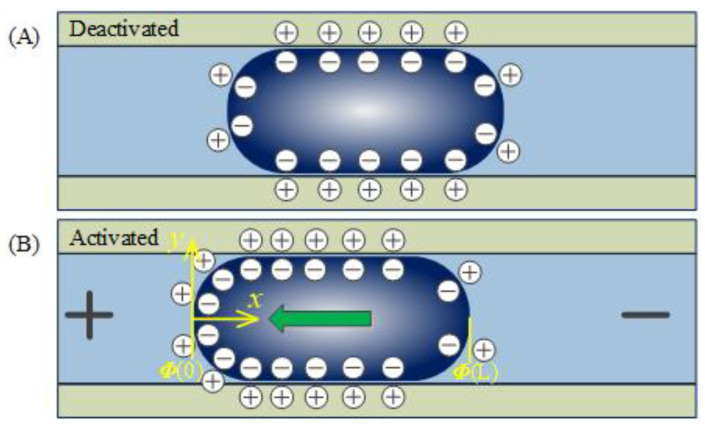
Working mechanism of the continuous electrowetting actuation of galinstan liquid metal. (**A**) Schematic of the charge distribution over the liquid metal surface when immersed in the NaOH solution. (**B**) Schematic of the surface charge distribution and transportation direction of the liquid metal upon the application of external electrical field.

It is well known that when a polarizable and conductive galinstan liquid metal slug is initially submerged in alkaline solutions such as NaOH electrolytes, the chemical reaction occurred between the liquid metal and the alkali solution, producing the anions [Ga(OH)_4_]^−^ at the liquid metal–electrolyte interface and making it negatively charged. Due to the ions’ absorption, the positive charges were attracted and accumulated at the interface to form the diffuse layer of the electrical double layer. Arising from the high conductivity of the liquid metal, the electric charge at the liquid metal-alkali solution interface could be seemed to be uniformly distributed, which could be deemed to be a charged capacitor, as illustrated in Figure 2. While with the electric field applied to the graphite electrodes at both ends, an external potential gradient could be produced along the channel (as shown in Figure 2B), causing the potential difference across the electrical double layer and therefore the electric charge redistribution above the liquid metal surface.

According to the thermodynamic analysis theory, in the interface, when the liquid metal was immersed in the electrolyte, the variation of the surface tension at the liquid metal–electrolyte interface with respect to potential difference between the liquid metal and electrolyte could be described by Young–Lippmann equation [46]:

(1) $$\gamma =\gamma_{0}-\frac{1}{2}c{\left(V-V_{0}\right)}^{2}$$

where, *γ* is the surface tension at the liquid metal-electrolyte interface, *γ*_0_ is the maximum value of the surface tension when no external electrical signal was applied. *c* and *V* are the capacitance per unit area for electrical double layer and potential difference across the electrical double layer, respectively. *V*_0_ is the intrinsic voltage across the electrical double layer. Meanwhile, *γ*_0_ is often deemed as the intrinsic surface tension generated by the chemical interactions over the interface of the neighboring immiscible fluids. According to Equation (1), the surface tension was affected by the electrical potential difference at the electrical double layer, which was believed to be an effective approach to induce local surface tension variation and was known as electrocapillarity.

Meanwhile, the electrical control of the surface tension was responsible for the pressure difference between the liquid metal and electrolyte interface, which was depicted by the Young–Laplace equation:

(2) $$\Delta p=\gamma \left(\frac{1}{r_{1}}+\frac{1}{r_{2}}\right)$$

where, Δ*p* is the pressure difference between the liquid metal and the electrolyte, and *r*_1_ and *r*_2_ are the principal curvature radii [47]. The contact angle could be defined to be approximately 180° when the liquid metal was in contact with aqueous liquid in the microchannel [42]. 

When the direct current (DC) bias was enforced on the graphite electrodes, the electrical double layer voltage was adjusted and a small current was generated between the liquid metal and the channel wall, resulting in less voltage drop near the cathode of the energized electrodes compared with the other side. The surface tension between the two immiscible conductive liquids decreased with the increasing electrical potential as described by the above equations. Hence, a higher surface tension occurred to the left side of the liquid metal slug where the surface tension was relatively lower, creating a local surface tension variation and causing the pressure imbalance between the left and right menisci of the liquid metal. Then, the liquid metal slug was induced to transfer from the cathode to the anode of the graphite electrode. This particular phenomenon of liquid metal movement driven by the electric field-induced surface tension gradient could be interpreted as the electrical Marangoni effect, in which the liquid metal with lower surface tension tended to wet more surrounding region than the higher menisci, to minimize its surface energy and the relative pressure differential, causing the movement of liquid metal slug. As long as the electrodes were energized by the electrical signal, the pressure difference would theoretically exist and therefore the liquid metal would be pumped continuously. The resultant phenomenon of liquid metal locomotion and the flow motion of the neighboring immiscible was defined as continuous electrowetting.

## 3. Results and Discussion

To explore the pumping performance of the proposed surface tension-induced device, we experimentally investigated the relationship between the actuation speeds of the liquid metal slug and the electrolyte concentration, voltage magnitudes and driving frequencies of the applied electric field, as well as the galinstan liquid metal droplet volume. 

### 3.1. Effect of Electrolyte Concentration

**Figure 3:**
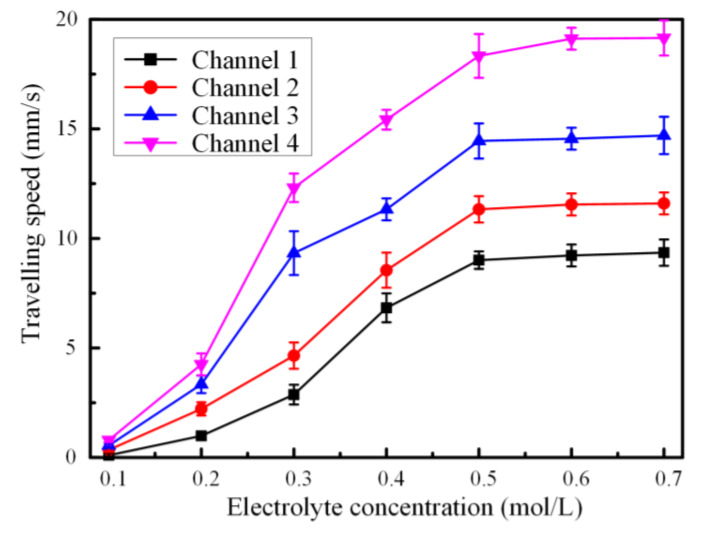
Plot of actuation speed of liquid metal slug with respect to the electrolyte concentration at a 150 Hz 4 Vpp sinusoidal wave with a 4 V DC offset.

As the chemical reaction occurred over the interface of liquid metal–electrolyte, the ion concentration in the electrical double layer would affect the pumping performance. As the chemical reaction occurred in the liquid metal–NaOH electrolyte interface, the oxide layer was removed to keep the continuously pumping [48]. Herein, we chose NaOH solution as the actuation medium and studied the pumping performance by changing the ion concentration of the NaOH solution ranging from 0.1 to 0.7 mol/L. As depicted in Figure 3, the travelling speed of the liquid metal slug increased the increment of the ion concentration, reaching to the maximum value of 19.15 mm/s when the ion concentration was 0.6 mol/L. Additionally, the saturation point was reached and the actuation speed varied slightly when the ion concentration was beyond 0.5 mol/L. It could be accountable that the initial charge in the electrical double layer increased with the ion concentration and the resultant pressure difference aggravated between the two hemispheres, which in turn improved the electrical actuation ability of the liquid metal slug in NaOH solution. Then, the initial charge became saturated with the increasing ion concentration, causing a slight difference in surface tension in the liquid metal–electrolyte interface. Additionally, it could be seen from the Figure 3 that the actuation speed enhanced with the shrinking of the electrode gap. We speculated that the narrow electrodes distance caused a smaller electrical resistance and the corresponding current would augment under the same circumstances. Eventually, the larger electrical actuation flow rate could be obtained due to the higher pressure difference caused by the longer electrical current path, which was in well accordance with the previous research [27].

### 3.2. Effect of Voltage Amplitude of Applied Electric Field

**Figure 4:**
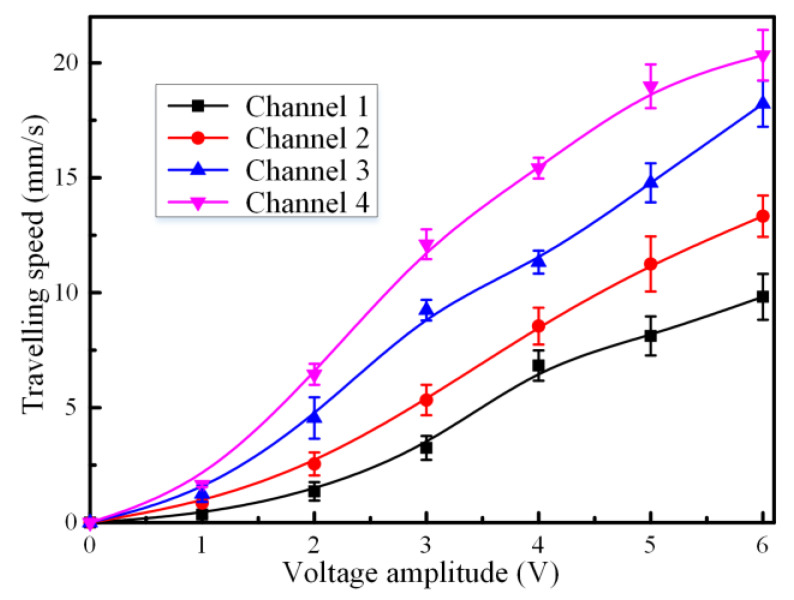
Plot of pumping velocity versus signal amplitude for liquid metal upon the application of a 150 Hz sinusoidal wave signal at 4 Vpp at the electrolyte concentration of 0.4mol/L.

It was presumably from the equation that the surface tension increased with the voltage magnitude of the exciting signal. We carried out a series of experiments to explore the changing rules of pumping velocity with respect to the voltage amplitude of the external electrical signal under different electrodes distances with a 150 Hz sinusoidal wave of 4 Vpp applied. The sinusoidal wave signal was utilized to avoid surface oxidation in the liquid metal–electrolyte interface. We observed the liquid metal moved toward the anode of the energized electrodes when the direct current was applied across the electrolyte. The liquid metal slug ceased once the voltage signal was removed. Meanwhile, the flow direction of liquid metal reversed instantly when the polarity of the applied direct current was shifted. We could also observe that the liquid metal slug moved back and forth at the equilibrium position by changing the polarity of the direct current electrical field. It could be seen from Figure 4 that the travelling speed of the liquid metal slug increased with the magnitude of the applied voltage. A maximum actuation speed of 20.33 mm/s was obtained with a driving voltage of 6 V DC offset at the NaOH concentration of 0.4 mol/L upon the application of a sinusoidal wave signal of 4 Vpp at 150 Hz. This was because the charge imbalance was generated, and the ions charges were redistributed in the electrical double layer upon the application of the external dynamical electrical field. Additionally, the electro-Marangoni effect was induced by the non-uniform surface tension arising from the electrical potential gradient. Neglecting the friction among the liquid metal, the glass substrate and PDMS channel, the electrical actuation speed was proportional to the voltage difference between the two ends of the liquid metal slug [42]. Therefore, faster movement of the liquid metal was obtained with the increasing electrical potential. However, an undesirable gas bubble was observed around the electrodes due to the electrolysis of the electrolyte solution at higher voltages. This was ascribed to the large current density arising from high voltage, which would result in generation of either hydrogen or oxygen in the vicinity of the driving electrodes. Another amazing phenomenon of stretching or even splitting of the liquid metal slug was observed at a high voltage such as 16 V. It was probably that the surface tension increased nonmonotonically with the applied electrical potential, making the surface tension dramatically augmented and finally breaking up the liquid metal slug. 

### 3.3. Effect of Galinstan Liquid Metal Volume

**Figure 5:**
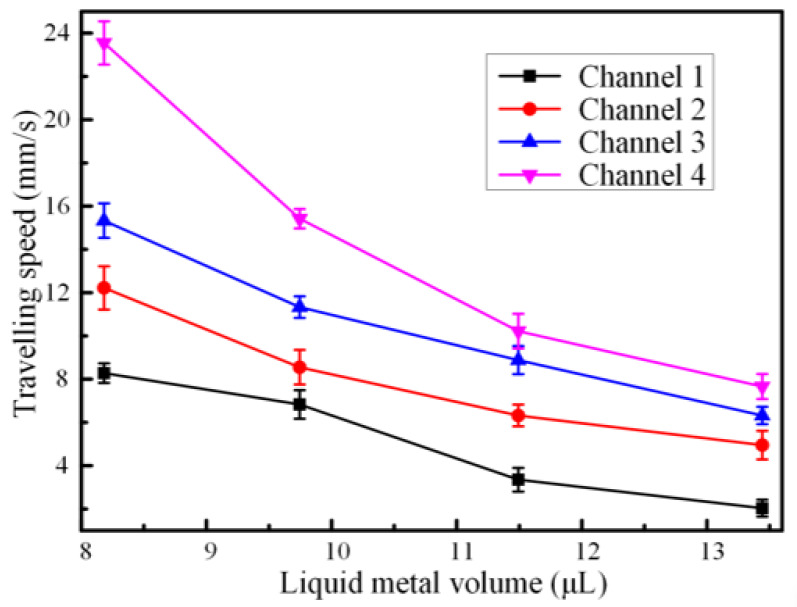
Plot of average actuation velocity versus liquid metal volume when a 150 Hz sinusoidal wave signal at 4 Vpp with 4 V DC offset was applied at the driving electrodes.

The length of the galinstan liquid metal slug could affect the travelling speed of the liquid metal in the channel with the rectangular cross section. We conducted a series of experiments to explore the electrical actuation performance by changing the diameter of the liquid metal. As shown in Figure 5, the larger liquid metal volume could lead to poor pumping performance, manifesting that the higher travelling speed could be obtained with smaller liquid metal droplets. A maximum speed of 23.55 mm/s was obtained with the liquid metal volume of 8.182 μL, while the neighboring exciting electrode distance was 8 mm. When the diameter of the liquid metal was small enough, the gap between the liquid metal and the channel would influence the electrostatic actuation. It was probably that less time was required for the ions in the electrical double layer to redistribute over the droplet surface when the droplet was smaller [27]. Additionally, it could be explained by the fact that the greater inertial force would be produced as the volume of liquid metal increased, indicating that a larger pressure difference was needed to move the liquid metal slug forward.

### 3.4. Effect of the Frequency of Alternating Current Electrical Field

**Figure 6:**
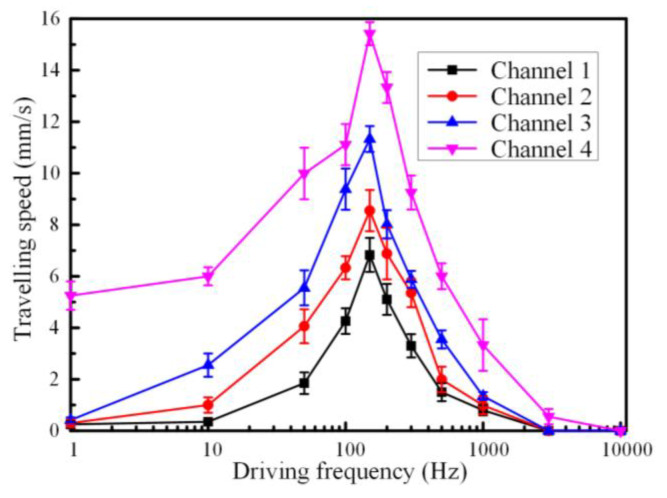
Plot of average actuation velocity versus different driving frequencies of the sinusoidal wave signal at 4 Vpp with 4 V DC offset when the ion concentration of NaOH was 0.4 mol/L.

As the sinusoidal wave alternating current signal was applied on the driving electrodes, we speculated that the driving frequencies of the alternating current may influence the electrostatic actuation ability. We conducted a series of experiments to investigate the pumping performance under different frequencies. The relationship between the flowrates of the liquid metal slug and the driving frequencies of alternating current electrical field was illustrated in Figure 6. On the whole, we could observe that the flowrate of the liquid metal slug increased with the frequency of the applied sinusoidal wave and the highest flowrate of approximately 16 mm/s was acquired when the frequency was set at around 150 Hz for channels with different electrode gaps, and then, the movement speed of the liquid metal decelerated dramatically with the increasing frequencies. There was no obvious liquid metal transportation when the driving frequencies were beyond 10 kHz. It could be possible that the liquid metal surface was gradually oxidized under low frequencies, causing the decrement in the pressure differences between the two neighboring hemispheres and slowdown of the pumping speed. Meanwhile, oscillating behavior was observed when the applied frequencies were below 20 Hz. The pumping performance was affected and was not obviously at the lower frequencies. While at high frequencies, the ions’ charge in the electrical double layer did not have enough time to redistribute before de-electrowetting [27]. Therefore, the oxidation effect occurring in the liquid metal–electrolyte interface seemed to be suppressed and the ions could be redistributed around 150 Hz, where the highest electrostatic actuation performance could be acquired.

**Figure 7:**
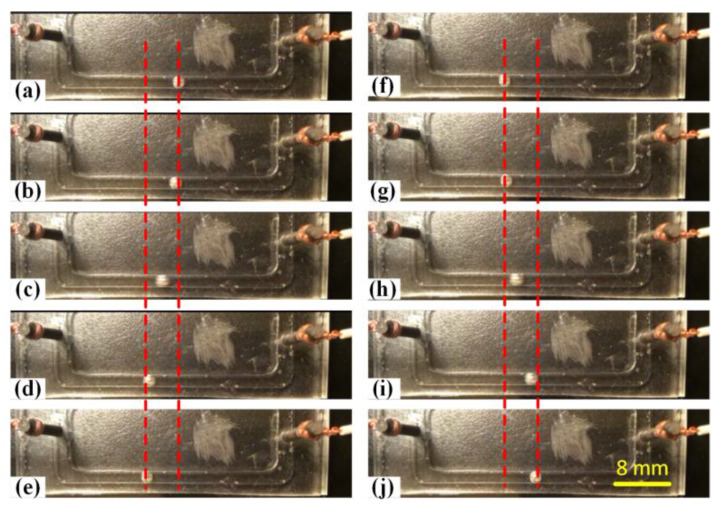
(**a**–**j**) Oscillation of the liquid metal droplet between two driving electrodes upon the application of a sinusoidal wave signal of 50 Vpp at 4 V DC offset at the frequency of 5 Hz.

Under most circumstances, we investigated the movement of the liquid metal slug in the alkaline solutions without considering the voltage polarity. In the previous section, with a relative smaller alternating current applying to prevent electrolysis, we found that the liquid metal would always tend to move onto the positive potential electrode when the electrodes were energized by direct current voltage. In addition, the liquid metal moved back and forth between the driving electrodes as the voltage polarity was switched. It seemed that the transportation modes of the liquid metal were polarity-dependent. Inspired by that, we experimentally explored the electrostatic actuation patterns of the liquid metal droplet at different resonance frequencies when a sinusoidal wave signal of 50 Vpp at 4 V offset was applied. Interestingly, we obviously observed that the liquid metal droplet moved back and forth regularly and continuously at the original position when the frequency was set to be below 10 Hz, resulting in the liquid metal droplet periodically oscillating between the two driving electrodes, which was depicted in Figure 7 and recorded in video S1. We also found that the oscillation frequency was determined by the frequency of the applied sinusoidal wave signal. It was speculated that the charge relaxing time may be affected by the applied AC frequency, while the liquid metal droplet’s hydrodynamic response was in the order of the AC driving frequency. With the polarity of the applied exciting signal shifting, the liquid metal droplet would oscillate periodically due to the change of the surface tension. The rapid oscillation behavior of the liquid metal droplet could be utilized to promote effective fluid mixing. 

**Figure 8:**
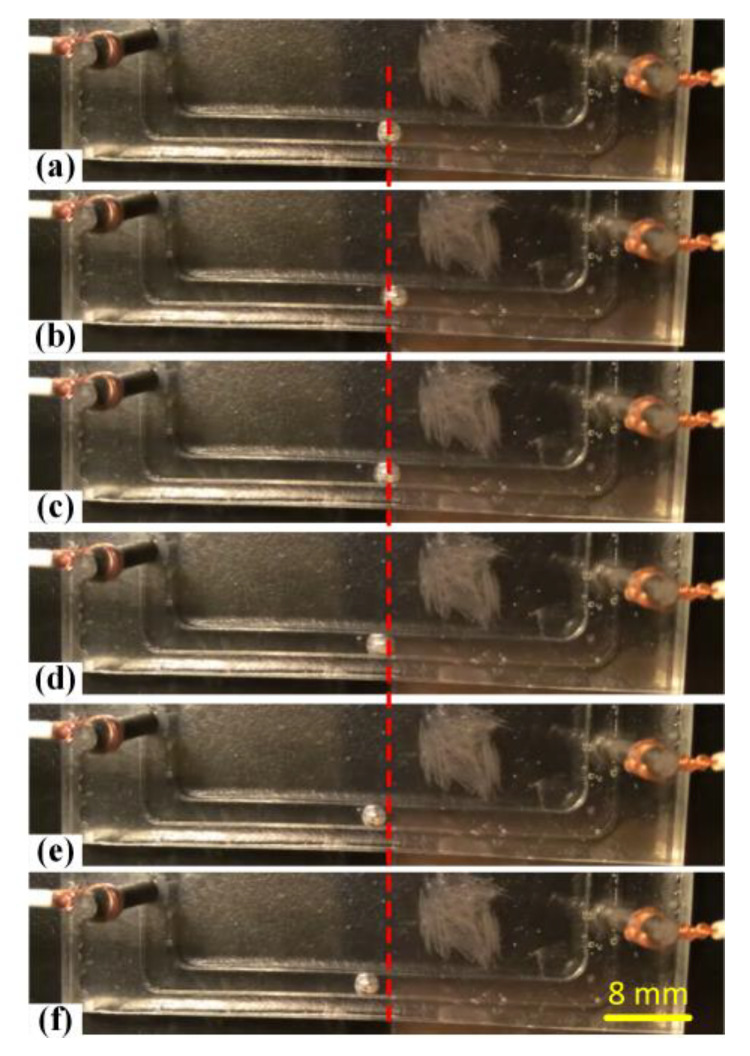
(**a**–**f**) Forward locomotion while oscillating of the liquid metal slug between two driving electrodes upon the application of a sinusoidal wave signal of 50 Vpp at 4 V DC offset at the frequency of 15 Hz.

With the increasing exciting frequencies, we could observe that the liquid metal droplet initially shook at the original position, accompanied with asymmetrical oscillating. Namely, the locomotion distance of the liquid metal droplet shaking to the left side was slightly bigger compared with the right side. Therefore, the pumping characteristics were exploited and the liquid metal could move toward the anode of the driving electrodes while oscillating when the applied frequency was in the low frequency range from 10 to 20 Hz, as described in Figure 8 and recorded in video S1. Under the same circumstances, we could also obviously observe that the oscillating magnitude of the liquid metal droplet was much smaller compared with the frequency below 10 Hz. This meant that the droplet oscillation was frequency-dependent and the oscillating magnitude decreased with the increasing frequency. The above phenomenon was in accordance with the previous reported results [44]. It was reported that the asymmetric droplet oscillation would induce a much chaotic fluid pattern than the symmetric oscillation and could be further exploited to enhance fluid mixing and fluid pumping in some droplet-based microfluidic devices.

**Figure 9:**
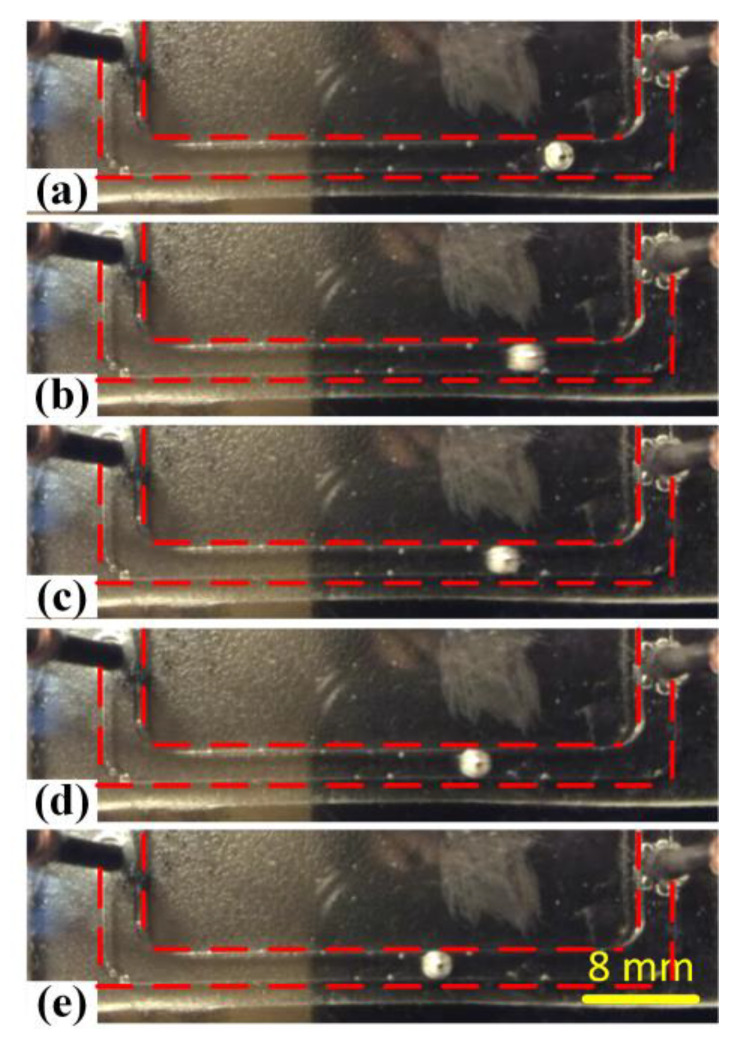
(**a**–**e**) Forward locomotion of the liquid metal slug between two driving electrodes upon the application of a sinusoidal wave signal of 50 Vpp at 4 V offset at the frequency of 30 Hz.

With no obvious droplet shape oscillation, the continuous forward locomotion of the liquid metal droplet toward the anode was obtained when the frequency was beyond 20 Hz. As shown in Figure 9, the continuous unidirectional pumping of the liquid metal droplet was observed upon the application of a sinusoidal wave signal of 50 Vpp at 4 V DC offset in response to a 30 Hz. The pumping characteristic was consistent with the experimental phenomenon mentioned above with the liquid metal slug. Meanwhile, the pumping speed was determined by the frequency of the sinusoidal wave signal. However, the undesirable bubbles were identified around the driving electrodes with the increasing frequency due to the electrothermal effect. 

## 4. Conclusions

In summary, we presented a nontoxic galinstan liquid metal-based microfluidic device based on continuous electrowetting. The pressure difference between the two hemispheres of the liquid metal slug was induced to move the liquid metal toward the anode of the electrodes due to the surface tension variation when the two neighboring graphite electrodes were energized by the external electric field. The parameters’ influences on the velocity of the liquid metal slug were experimentally investigated. The results showed that the travelling speed of the liquid metal increased with the concentration of the filling electrolytes and the applied voltage magnitude. With the increasing frequencies, the translation velocity of the liquid metal increased to a certain value and then decreased. Meanwhile, the speed of the liquid metal slug diminished with the droplet size. The transportation speed of the liquid metal slug in the 0.4 mol/L NaOH concentration was 20.33 mm/s when the graphite electrode was activated by a sinusoidal wave signal of 4 Vpp at 150 Hz at 6 V DC offset. The splitting and stretching phenomenon of the liquid metal slug occurred at a high voltage such as 16 V. Additionally, we further investigated the motion dependency of the liquid metal under the low frequencies, and demonstrated two other movement patterns, including oscillation and forward while oscillating. Future research is required to integrate the micro-size liquid metal into the micro-actuator, which may enable it to be utilized in microelectronic devices. The highly controllable actuating behavior of electric field-induced liquid metal locomotion made it feasible in various applications of the MEMS actuator, micro heat-exchanger, intelligent soft robot and reconfigurable electronic structures. 

## Supplementary Materials

The following are available online at https://www.mdpi.com/1996-1944/13/9/2122/s1, Video S1: Versatile movements of the liquid metal under electrostatic actuation.
